# Placenta Accreta Spectrum (PAS): Diagnosis, Clinical Presentation, Therapeutic Approaches, and Clinical Outcomes

**DOI:** 10.3390/medicina60071180

**Published:** 2024-07-20

**Authors:** Filiz Markfeld Erol, Johanna Alena Häußler, Markus Medl, Ingolf Juhasz-Boess, Mirjam Kunze

**Affiliations:** Department of Obstetrics and Gynecology, Medical Center, University Hospital Freiburg, 79106 Freiburg, Germanymarkus.medl@uniklinik-freiburg.de (M.M.);

**Keywords:** placenta praevia, abnormal adhesion, cesarean delivery, server peripartum bleeding

## Abstract

Placenta accreta spectrum (PAS) refers to the abnormal adhesion of the placenta to the myometrium, with varying degrees of severity. Placenta accreta involves adhesion to the myometrium, placenta increta invades the myometrium, and placenta percreta extends through the serosa to adjacent organs. The condition is linked to deficient decidualization in scarred uterine tissue, and the risk increases when placenta previa is present and with each prior cesarean delivery. Other risk factors include advanced maternal age, IVF, short intervals between cesareans, and smoking. PAS incidence has risen due to the increase in cesarean deliveries. Placenta previa combined with PAS significantly raises the risk of severe peripartum bleeding, often necessitating a cesarean section with a total hysterectomy. Recognizing PAS prepartum is essential, with sonographic indicators including intraplacental lacunae and uterovesical hypervascularization. However, PAS can be present without sonographic signs, making clinical risk factors crucial for diagnosis. Effective management requires a multidisciplinary approach and proper infrastructure. This presentation covers PAS cases treated at University Hospital Freiburg, detailing patient conditions, diagnostic methods, treatments and outcomes.

## 1. Introduction

**Figure 1:**
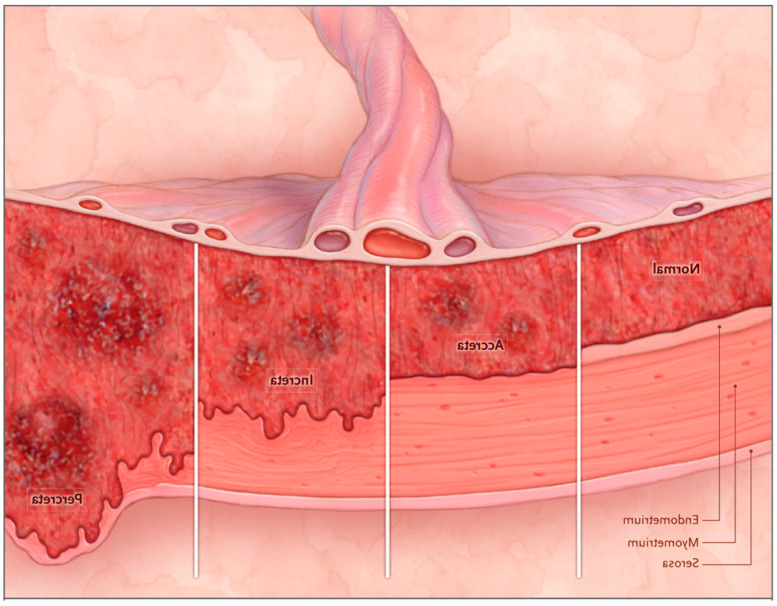
Schematic presentation of PAS. Silver and Branch NEJM April 2018. Placenta accreta: attachment of the placenta to the myometrium without an intervening decidua), Placenta increta: invasion of the trophoblast into the myometrium, Placenta percreta: invasion through the myometrium, serosa, and surrounding structures.

Placenta accreta spectrum (PAS) is a term used to describe a pathological condition characterized by abnormal adhesion of the placenta to the myometrium of the uterus. The severity of the invasion can vary widely, hence the term “spectrum.” The most common form of PAS is placenta accreta, which involves abnormal placental adhesion to the myometrium. Placenta increta occurs when there is invasion of the myometrium, and the most severe form is placenta percreta, which involves invasion through the serosa surrounding the uterus and may extend to adjacent organs such as the bladder, intestines, and parametrial ligaments (Figure 1) [1,2,3].

The etiology of PAS is likely related to deficient decidualization of the endometrium in areas of scarring within the uterine tissue. This leads to abnormal attachment of the chorionic villi to the myometrium or infiltration of the trophoblast [4].

Therefore, the risk of developing placenta accreta spectrum (PAS) is heightened when placenta previa is detected, with incidence rates ranging from 11% to 25%. Furthermore, the risk increases significantly with each prior cesarean delivery, with studies indicating an elevated risk of 5% to 10% per previous cesarean [5,6,7,8,9,10].

Other interventions within the uterus that damage the endometrium, particularly those involving the opening of the uterine cavity, are also associated with an increased risk of placental disorders. These interventions include curettages and endometrial ablations. Additional risk factors for placental disorders include advanced maternal age, pregnancy following in vitro fertilization (IVF), a short interval between cesarean delivery and subsequent pregnancy, and smoking [11].

The incidence of deficient placentation resulting in placenta accreta spectrum (PAS) has risen from 1 in 2510 to 4017 births to 1 in 533 births. This increase is attributed to the higher prevalence of cesarean deliveries, which subsequently leads to a greater number of PAS cases in subsequent pregnancies [12,13].

The combination of placenta previa and placenta accreta spectrum (PAS) poses a particularly high risk of massive peripartum bleeding. This can lead to complex postpartum courses characterized by significant maternal morbidity and mortality.

In most cases involving placenta accreta spectrum (PAS) and severe peripartum bleeding, a cesarean section combined with a total hysterectomy—one of the most challenging operations in obstetrics—becomes inevitable.

To effectively manage high-risk pregnancies, it is crucial to recognize prepartum signs of placenta accreta spectrum (PAS). Sonographic findings indicative of PAS include intraplacental lacunae, loss of the hypoechogenic retroplacental (clear) zone, an irregular barrier between the uterus and bladder, interruption between the uterus and bladder (bright bladder line), or exophytic tumors within the bladder. Additionally, colored Doppler sonography may reveal uterovesical hypervascularization, subplacental hypervascularization, bridging vessels (vessels crossing the barrier between myometrium and placenta), or feeder vessels supplying placental lacunae.

Even in the absence of pathological findings on sonographic examination, the possibility of PAS cannot be ruled out entirely. Thus, it is essential to consider clinical risk factors. Early prepartum diagnosis is crucial for improving outcomes. Optimal management involves a standardized approach with a multidisciplinary team and appropriate infrastructure, such as access to intensive care units and blood management services.

This paper highlights cases of patients diagnosed with placenta accreta spectrum (PAS) who received treatment at University Hospital Freiburg, Germany. Each case provides a detailed overview of the patient’s condition, diagnostic approach used, treatment strategy, and outcomes.

## 2. Case 1

A 36-year-old woman, in her fourth pregnancy (Gravida IV/Para I), presented at 37 weeks gestation with placenta previa. The patient had previously undergone a secondary cesarean section with a T-incision at an external hospital.

**Figure 2:**
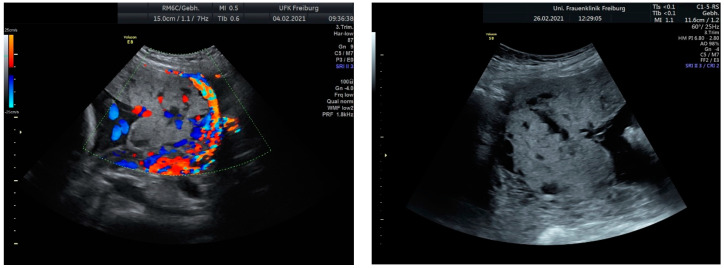
Irregular and large lacunae: Sonographic imaging reveals irregularly shaped and enlarged lacunae within the placenta. Hypervascularity: Increased vascularity is observed within the placenta, indicating abnormal blood flow. Turbulent flow in lacunae: Doppler imaging demonstrates turbulent blood flow within the lacunae, suggestive of abnormal vascularization. Changes in diameter: Variations in the diameter of lacunae may be noted on sonographic examination, reflecting the dynamic nature of placental vascularization in PAS.

Sonographic findings (Figure 2):

After extensive consultations, we reached a consensus to honor the patient’s preference for uterine preservation where feasible. Following a multidisciplinary case discussion, we scheduled a cesarean section at 37 + 0 weeks and prepared multiple units of erythrocyte concentrates. Prior to commencing the procedure, a cell saver system was set up.

The uterotomy was performed as high as possible. The newborn presented in a torso position and was successfully delivered after converting them to a foot presentation. Following umbilical cord clamping, meticulous manual dissection of the placenta was carried out. The placenta was found to be adherent, showing features of accreta and, in some areas, increta involving the frontal aspect of the uterus.

Following curettage of the uterine cavity, there was a sudden escalation of bleeding originating from the lower uterine segment. Abdominal packing was employed to control the hemorrhage, followed by suturing of the bleeding vessels. Diagonal stitches were applied, along with the insertion of a Bakri balloon. The uterotomy was closed using a continuous suture technique without encountering further complications.

Throughout the procedure, the patient received four units of erythrocyte concentrates, four doses of fresh frozen plasma (FFP), 2 g of tranexamic acid, and 4 g of fibrinogen to manage hemostasis effectively.

**Figure 3:**
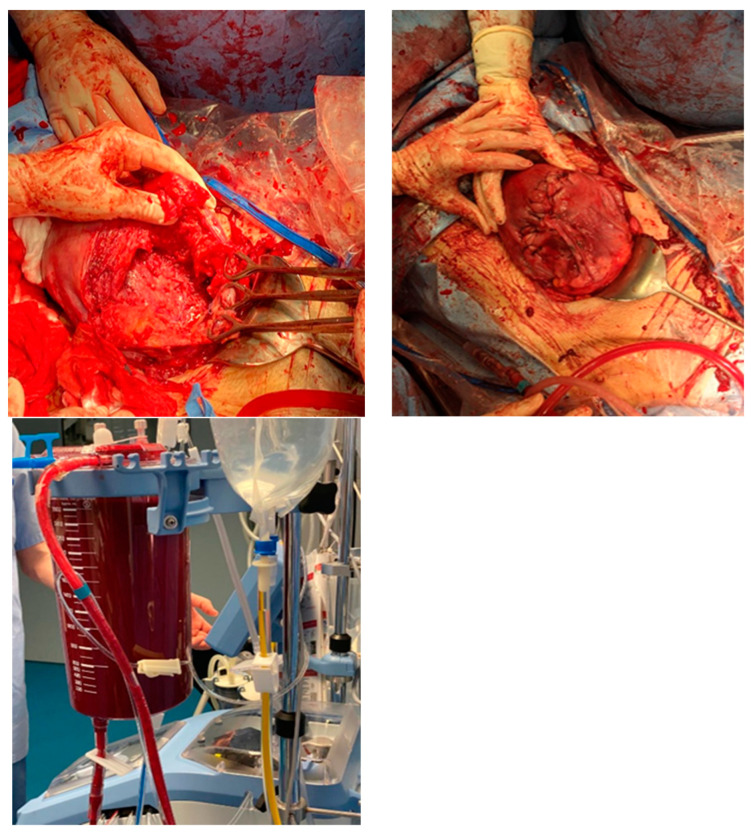
Intraoperative photodocumentation of the uterus and cell saver system. During the postoperative assessment, we identified anemia with a hemoglobin level of 6.0 g/dL. The patient was administered another unit of red blood cells. The Bakri balloon was removed the next day without any complications. The patient was discharged in good general condition with a hemoglobin level of 7.4 g/dL.

Intraoperative findings (Figure 3): 

## 3. Case 2

A 27-year-old woman (G3P2) at 37 + 5 weeks gestation, having had two previous cesarean deliveries, presented with placenta previa originating from the anterior wall of the uterus.

**Figure 4:**
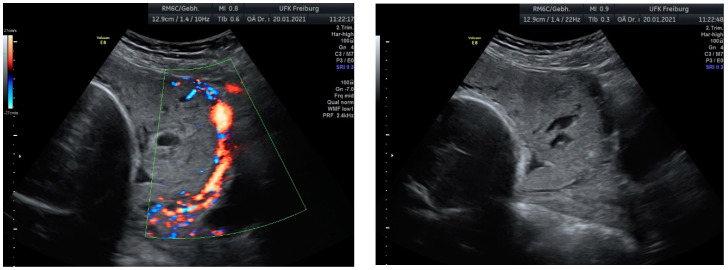

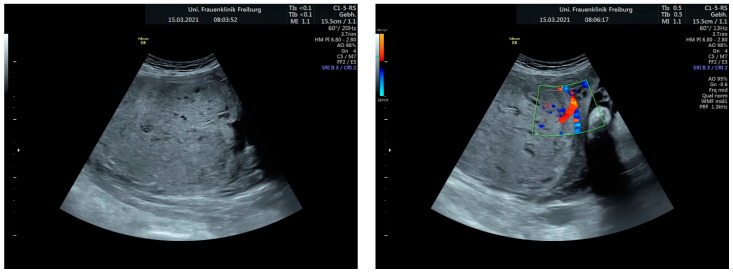
shows suspected placenta increta with dehiscence in the area of scar tissue from the previous uterotomy.

**Figure 5:**
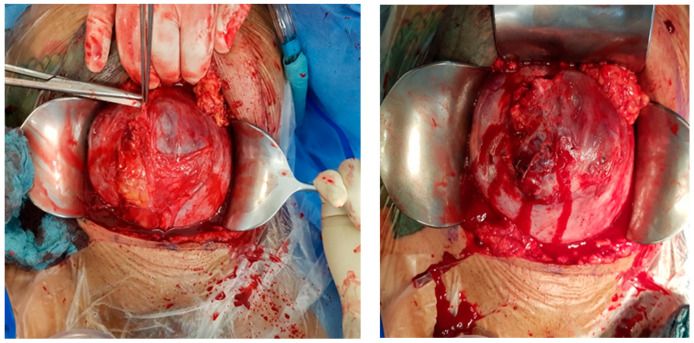
Reveal placenta previa with placenta increta. The earlier suspected dehiscence was confirmed.

Sonographic findings (Figure 4): 

Intraoperative findings (Figure 5): 

Following resection of the anterior uterine wall, a Bakri balloon was inserted, and the anterior wall was reconstructed using single-button sutures. The patient received 5 units of erythrocyte concentrates, 4 units of fresh frozen plasma (FFP), 2 g of tranexamic acid, and 24 micrograms of Desmopressin.

Postoperatively, the patient was transferred to the intensive care unit and administered antibiotic treatment consisting of cefazolin 2 g (1-0-1) and metronidazole 500 mg (1-0-0) for three days. Subsequently, due to persistent fever, the antibiotic regimen was changed to meropenem.

On the seventh day post-operation, the patient was discharged in a good condition with a hemoglobin level of 7.1 g/dL.

## 4. Case 3 

A 37-year-old woman (G2P1) at 15 + 2 weeks gestation with dichorionic–diamniotic twins conceived through assisted reproductive technology (ICSI) presented to the emergency room. She had a history of a previous emergency cesarean section due to preterm premature rupture of membranes (PPROMs) at 36 + 1 weeks, complicated by velamentous cord insertion, resulting in the loss of her first child. The patient presented with abdominal pain and signs of hemorrhagic shock.

Upon examination, free fluid was detected in the abdomen, raising suspicion of uterine rupture. Despite the critical condition, the patient expressed a desire to preserve the pregnancy if feasible.

During surgery, a confirmed rupture of the uterus was identified—a 3 cm fissure within the scar tissue from the previous uterotomy, with a small piece of placenta protruding from the fissure. This 4 mm segment of placental tissue was removed, and the fissure was meticulously sutured under sonographic guidance, successfully stopping the bleeding.

Given the significant risk of recurrent rupture during the ongoing pregnancy, termination of the pregnancy was deemed medically advisable. However, the patient had not provided consent for termination prior to the operation, and since the bleeding was controlled post-repair, further interventions were withheld after placement of a drainage system.

**Figure 6:**
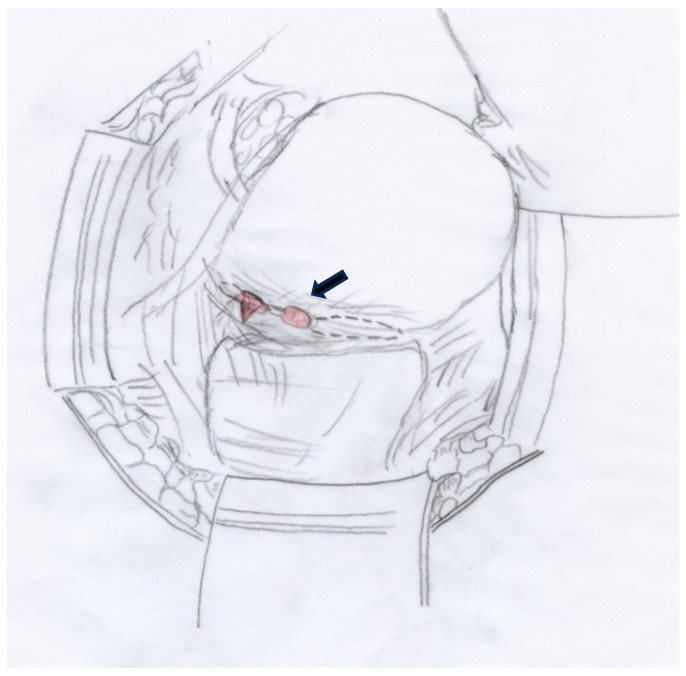
Schematic drawing illustrating the small uterine rupture with a portion of placental tissue protruding from the rupture site, which was the source of bleeding.

During the procedure, the patient received two units of packed red blood cells due to intraoperative blood loss (Figure 6).

Following the operation, the patient was monitored in the intensive care unit for one day. She received an additional three units of packed red blood cells and commenced on antibiotic therapy with Cefuroxime 1.5 g (1-1-1). Due to a persistent fever (up to 38.8 °C), the antibiotic regimen was escalated to Piperacillin/Tazobactam and Metronidazole, resulting in a rapid clinical improvement.

The patient experienced symptoms suggestive of intestinal obstruction, which were managed collaboratively: administration of prokinetic medication and placement of a gastric tube led to a prompt resolution of symptoms.

Subsequent vaginal swab results identified Candida albicans, Enterobacter cloacae, and E. coli, prompting treatment with Fluconazole (oral and local) and Ciprofloxacin.

In the ensuing days, no further complications arose. Extensive discussions were held with the patient and her partner to explain the associated risks (maternal and fetal) and the prognosis of the ongoing pregnancy. Despite the challenges, the patient opted to proceed with the pregnancy and was discharged with viable twins on the 18th day following the operation.

**Figure 7:**
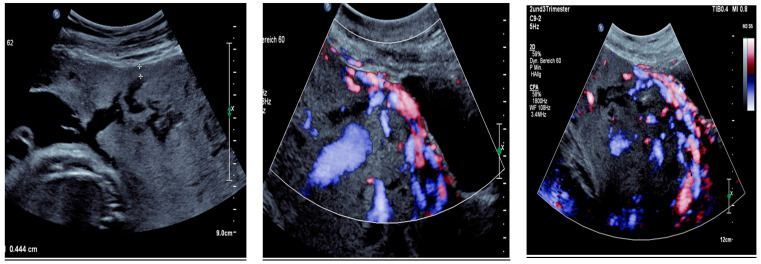
Revealed the following sonographic findings:

Sonographic findings (Figure 7): 

A very small barrier between the uterus and the bladder, with no recognizable

- myometrium in this area.
- Numerous blood vessels, most parallel and two perpendicular.
- Multiple placental lacunae confirmed by color Doppler.

Based on these findings, we planned an elective cesarean section at 35 weeks of gestation, combined with a hysterectomy due to the confirmed placenta increta and the high risk of significant bleeding. These findings were confirmed during the laparotomy.

During the procedure, both twins were born without complications. The uterotomy was closed, and the uterus was completely removed with the placenta in situ. The patient received four units of packed red blood cells, four doses of fresh frozen plasma (FFP), 3 g of tranexamic acid, and 28 micrograms of Desmopressin to manage bleeding and maintain hemostasis.

**Figure 8:**
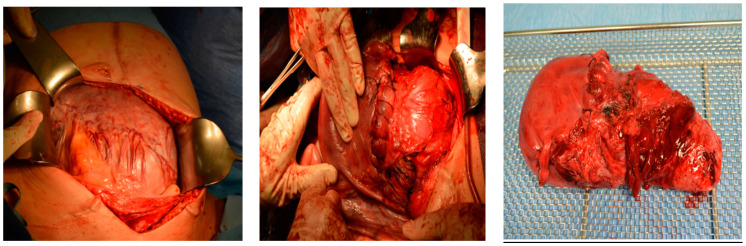
Depict the uterus during and after hysterectomy performed for placenta increta.

Intraoperative findings (Figure 8): 

Histological findings of the uterus weighing 939 g revealed the following:

- Parts of a placenta increta located close to the sutured ruptured area.
- Absence of decidua basalis in the affected area, with focal necroses visible.
- The rest of the serosa showed no additional pathological findings.

## 5. Case 4

A 39-year-old woman (G8P3) presented at 35 + 2 weeks gestation, with a history of two previous cesarean deliveries and five miscarriages requiring four curettage procedures. She also had gestational diabetes managed with dietary interventions. The placenta was diagnosed as placenta previa totalis, originating from the anterior wall of the uterus. Placenta accreta spectrum (PAS) was suspected based on sonographic findings, including a small barrier between the bladder and uterus, bridging vessels, and placental lacunae.

**Figure 9:**
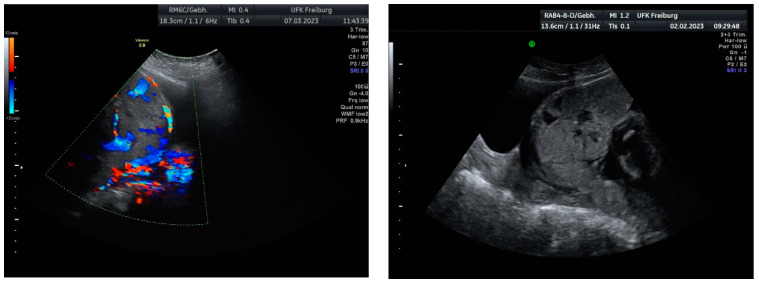
Irregular, large lacunae within the placenta, Hypervascularity, Turbulent flow inside the lacunae Diameter gaps.

Sonographic findings (Figure 9): 

Due to a strong suspicion of placenta accreta/increta and the completion of family planning, the patient underwent a hysterectomy and bilateral salpingectomy, including removal of the cervix. Placenta accreta spectrum (PAS) was confirmed during the procedure, and an abdominal drain was placed.

Following surgery, when the hemoglobin level dropped to 7.5 g/dL, the patient received Iron (III) hydroxide polymaltose complex to address iron deficiency. With a further decline in hemoglobin to 6.0 g/dL, the patient received two units of packed red blood cells. Tranexamic acid was administered twice to manage bleeding. The hemoglobin level subsequently increased to 8.7 g/dL and stabilized at 7.9 g/dL upon discharge in a good condition.

After seven days (duration not specified), the patient was readmitted due to severe upper abdominal pain and elevated inflammatory markers. A revision was performed for suspected infected hematoma at the vaginal stump, and antibiotic therapy was initiated. The patient was discharged on the fourth postoperative day in a good condition.

**Figure 10:**
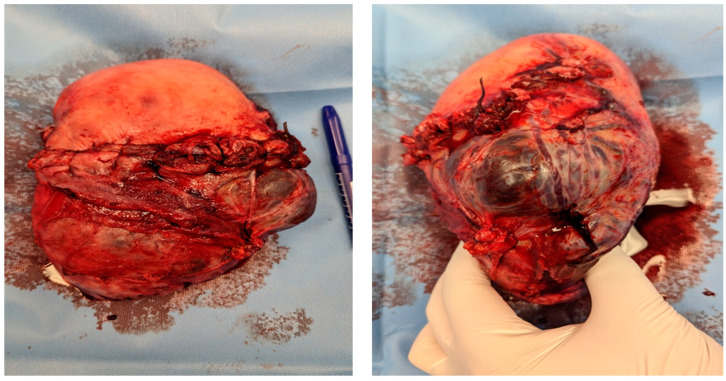
depict the uterus after hysterectomy with placenta previa and increta extending to the serosa. These images show the following findings:

Intraoperative findings (Figure 10):

- The uterus following a hysterectomy illustrates the presence of placenta previa with increta extending to the outermost layer of the uterine wall (serosa).

This confirms the suspected diagnosis of placenta previa with placenta increta, which necessitated the hysterectomy procedure to manage the condition.

Histological findings:

- Uterus with mature placenta accreta et praevia totalis
- Myometrium and serosa unremarkable. Endocervical retention cysts (ventral and dorsal). Including inconspicuous membranes and a three-vessel umbilical cord
- Inconspicuous resection margins towards the cervical ventral and dorsal, towards the bladder and rectal pillars, and on the right and left parametria.

## 6. Case 5

A 40-year-old woman (G7P2) presented at 38 weeks gestation, with a history of two previous vaginal deliveries, three abortions with dilation and curettage (abrasio), and an ectopic pregnancy (EUG). Placenta previa totalis with posterior uterine insertion was diagnosed early in the pregnancy. Placenta accreta spectrum (PAS) grade II was suspected, and the patient had completed her family planning. The fetus was in a transverse position, requiring planned cesarean delivery with comprehensive preparations.

During the delivery, the placenta was carefully assessed. It was observed that the placenta had invaded into the lower posterior uterine wall, extending into the serosa. Detachment without removing parts of the uterus was deemed impossible due to severe bleeding, necessitating an immediate hysterectomy.

At the time of the hysterectomy, 2000 mL of blood was collected in the Cell Saver system, and the patient began to exhibit signs of hemorrhagic shock. Following stabilization under anesthesia, the patient received a transfusion of packed red blood cells. The blood collected in the Cell Saver was processed and 1800 mL of washed blood was transfused back to the patient.

Postoperatively, the patient was monitored in the intensive care unit for 2 days and discharged on the seventh postoperative day in good condition with a hemoglobin level of 7.4 g/dL.

**Figure 11:**
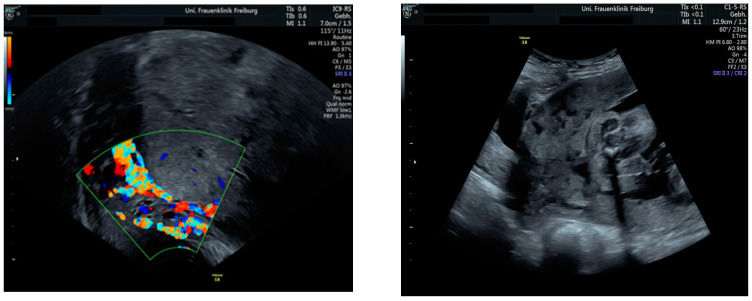
Increased placental blood flow on 2D ultrasound. Red color represents increased placental blood flow moving to the transducer; blue color represents placental blood flow moving leaving away from the transducer.

Sonographic findings (Figure 11): 

Histological findings:

- Hysterectomy specimen, 676.0 g, with parts of a placenta growing into the myometrium. Placenta growing into the myometrium, also extending directly to the ventral periuterine connective tissue.
- So-called placenta increta and also percreta with reduced decidua in the region of the decidua in the region of the fimbrial suture.
- Tubal and fimbrial funnels with edematous stroma. Inconspicuous fallopian tube.

## 7. Case 6

A 35-year-old woman (G3P1) at 34 + 1 weeks gestation presented with placenta previa totalis. She had a history of a previous cesarean section due to breech presentation and preterm premature rupture of membranes (PPROMs). The first cesarean section had revealed a uterine anomaly suggestive of a uterine duplex or complete uterine septum. Additionally, the patient had undergone dilation and curettage (abrasion) in the past.

The planned cesarean section at 35 + 0 weeks was expedited due to vaginal bleeding. Intraoperatively, it was discovered that the patient had placenta increta to percreta, where the placenta was deeply embedded into the uterine wall.

Following the delivery of the child under spinal anesthesia, a total hysterectomy with salpingectomy was performed under general anesthesia to manage the placental complications. The patient required two units of packed red blood cells during the operation and was monitored in the intensive care unit (ICU) for two days postoperatively.

Upon stabilization, the patient was discharged on the fifth day in good condition with a hemoglobin level of 8.0 g/dL.

**Figure 12:**
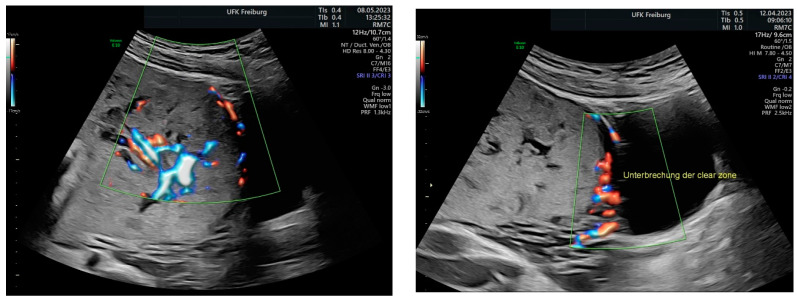
Enlarged vascular spaces within the placenta; A focal mass invading the myometrium, suggestive of placenta accreta; Hypervascularity noted at the interface between the serosa (outer uterine layer) and bladder.

Sonographic findings (Figure 12): 

These findings are characteristic of placenta accreta, a condition where the placenta attaches too deeply into the uterine wall, potentially leading to complications such as excessive bleeding during delivery. The hypervascularity at the serosa–bladder interface indicates the abnormal and increased blood flow associated with this condition. These imaging findings are crucial for diagnosis and guiding management decisions in cases of placenta accreta.

Histological findings:

- Placenta praevia totalis with regressively altered placenta at the cervix–corpus junction.
- Mild chronic endocervicitis with microglandular hyperplasia and transition to mature squamous epithelium of the ectocervix.
- Mature squamous epithelium of ectocervix ventrally and dorsally. Normal Myometrium and abundant decidualized endometrium. Inconspicuous serosa.
- Inconspicuous resection margins of the ectocervix ventral and dorsal, of the parametria as well as the rectal and bladder columns.

## 8. Case 7

A 35-year-old woman (G3P2) at 33 + 3 weeks gestation presented with placenta previa totalis located at the frontal wall of the uterus. The patient had a history of two previous cesarean deliveries.

Prior to the acute event, multiple sonographic findings raised the suspicion of placenta accreta spectrum (PAS), including loss of the clear zone, abnormal placental lacunae, myometrial thinning, and hypervascularization in the subplacental zone. An elective cesarean section was planned at 37 weeks due to these findings.

However, at 33 + 3 weeks, the patient was urgently transported via helicopter due to significant vaginal bleeding. Upon arrival, she was in a stable condition and had already received tranexamic acid. During the attempt to administer spinal anesthesia, the patient experienced sudden severe abdominal pain and increased vaginal bleeding, prompting immediate concern for uterine rupture. General anesthesia was induced, and an emergency cesarean section was performed, confirming the suspected uterine rupture during the procedure.

The placenta was found to have infiltrated the frontal wall of the uterus, specifically in the area of the previous cesarean scar, extending into the bladder wall—a condition consistent with placenta increta. Given the circumstances of placenta increta, uterine rupture, and the patient’s definitive family planning, a decision was made to proceed with a hysterectomy. The hysterectomy was performed without complications.

Postoperatively, the patient was transferred to an intensive care unit for two days and received four units of packed red blood cells due to significant blood loss. Seven days after the operation, she was discharged in good general condition, having successfully managed this complex obstetric situation.

**Figure 13:**
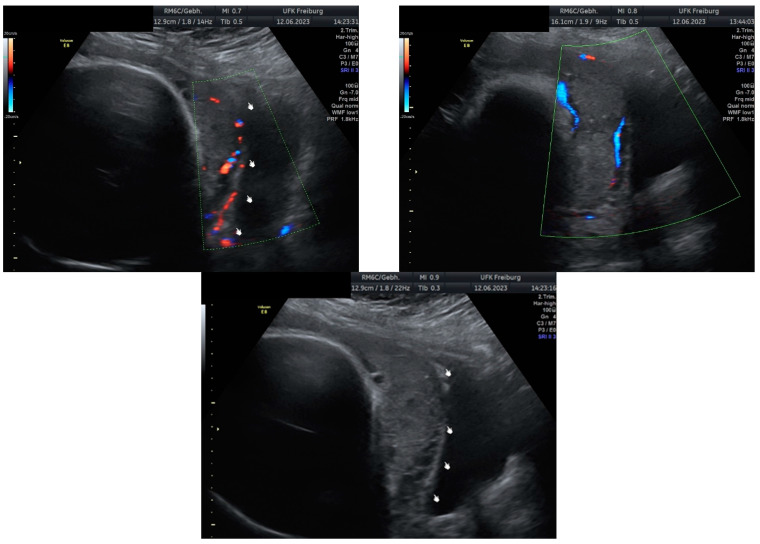
According to the standardized description of the European Working Group on Abnormally Invasive Placenta for ultrasound anomalies, the following anomalies may be seen: Loss of the “clear zone”—partially more in the middle (N/3):

Sonographic findings (Figure 13):

- Abnormal placental lacunae;
- No interruption of the echoic bladder wall Myometrial thinning;
- No protrusion of the placenta into the bladder wall;
- No uterovesical hypervascularization;
- Subplacental hypervascularization.

Histological findings:

- Hysterectomy specimen showing evidence of extravillous trophoblasts within decidualized endometrium decidualized endometrium and in the myometrium.
- Inconspicuous serosa. Unremarkable ventral and dorsal resection margins. Inconspicuous resection margins of the parametria
- The morphologic and histologic findings are consistent with placenta praevia and accreta.

## 9. Case 8 

A 32-year-old woman (G2P1) at presented 38 + 6 weeks gestation, having undergone a cesarean section with atony and B-Lynch sutures in first pregnancy. Because of the history of B-Lynch sutures, the mother desired a repeat cesarean.

**Figure 14:**
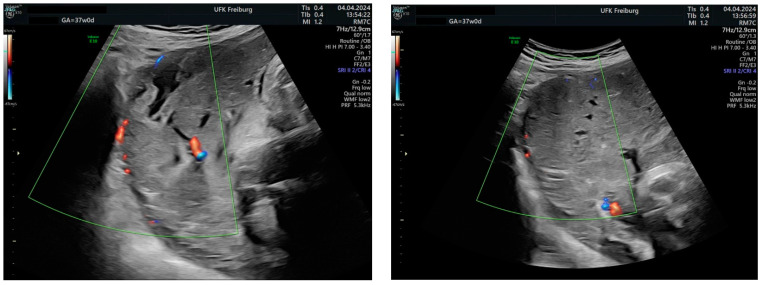
Separation from uterus is unclear on the right lateral wall and posterior wall, and so PAS cannot be excluded.

**Figure 15:**
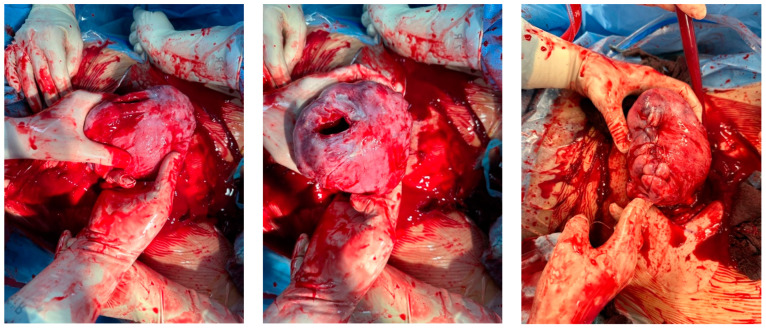
An approximately 5 cm fundal wall defect at the site suspected from the ultrasound after removal of the increta placenta; Suturing the defect with sutures without increased bleeding.

Sonographic findings (Figure 14): 

Intraoperative Findings (Figure 15):

The placenta was adherent to the right-side wall. After manual dissection, an open uterine wall defect of more than 5 cm was found at the fundus. This was closed with multiple single-button sutures. The patient received Sulproston and tranexamic acid in case of increased bleeding. Postoperatively, the patient presented with anemia of 6.4 g/dL and was started on RBC concentrate and Iron (III) sodium D-gluconate complex intravenously. This resulted in an adequate increase in Hb. The puerperium was uneventful. Postpartum sonography showed that the kidneys were not obstructed bilaterally and the uterine cavity was linear. The wounds were dry and without irritation. 

Due to the presence of placenta percreta and a defect in the right uterine wall, we advised the patient to deliver the pregnancy at 24 weeks at the earliest and to undergo a primary resection on her uterus without contractions.

## 10. Discussion

The early diagnosis of placental disorders by ultrasound allows the necessary measures to be taken and serious bleeding complications to be avoided. Several ultrasound features can lead to the diagnosis of placental disorders.

For the standardized definition of ultrasound markers, the European Working Group on Abnormally Invasive Placenta (EW-AIP.org.) was established to standardize the definition of ultrasound markers. The goal of this working group, which consists of 29 experts from 11 European countries, is to improve and simplify the diagnostic criteria for PAS [13].

Ultrasound criteria for PAS:

- Irregular and large intraplacental lacunae with turbulent flow in the absence of decidua;
- Abnormalities of the uterovesical interface: disrupted interface;
- Hyperechoic zone between the uterine serosa and the bladder wall (“bright bladder line”);
- Loss of hypoechoic retroplacental zone (“clear zone”);
- Exophytic masses extending into the bladder (Percreta);
- Myometrial thickness < 1 mm and uterine serosal protrusion.

The most important 2D color Doppler signs for PAS:

- Uterovesical hypervascularization: Significantly increased color Doppler signal between the myometrium and the posterior bladder wall. Numerous branching vascular bundles with multidirectional flow pattern and aliasing artifact.
- “Subplacental hypervascularization”: Significantly increased blood flow signal in the placental bed.
- “Bridging vessels”: Vessels originating from the placenta, usually running vertically through the myometrium and possibly through the serosa into the urinary bladder, ureter, or other organs.
- Placental lacunae feeder vessels: Vessels with a high flow velocity (>15 cm/s) that extend from the myometrium into the placental lacunae with turbulent flow patterns.

Even in the absence of ultrasound abnormalities, especially in the presence of risk factors, a placental disorder as defined by PAS may be present. Ultrasound findings are subject to a high interobserver variability. In case of PAS delivery, only in a level I perinatal center with an attached blood bank and an experienced team, if HE is necessary, urological stand-by, cell saver if necessary.

Magnetic resonance imaging may also be used, especially to evaluate posterior placentas and extension into adjacent organs. Several studies have compared ultrasound and MRI for the diagnosis of PAS [14,15,16]. Despite the heterogeneity of the studies and the results obtained, MRI is defined as a non-essential method in the diagnosis of PAS.

If the placenta is suspected of growing into the bladder, cystoscopy is recommended.

The most common treatment for high-grade placental abnormalities is a cesarean hysterectomy. In addition to the loss of fertility and the psychological consequences of a hysterectomy, it is associated with high maternal morbidity [17,18]. It can also result in significant blood loss with associated complications such as the need for blood transfusions or the occurrence of disseminated intravascular coagulation [18].

In a conservative approach, only the placenta or affected tissue is removed. This was possible in cases 1, 2, and 3. After surgical removal of the PAS areas, the defect can be reconstructed [2]. For larger placental adhesions, a generous en bloc resection is advisable [19]. After removal and hemostasis, a Postpartum Balloon with Rapid Instillation Component scan [20] can be used for smaller areas. A wait-and-see approach, leaving the placenta in the cavity after development and separation of the baby, is experimental [17] and requires careful monitoring of the patient as it may be associated with high maternal morbidity [21]. Success depends on the degree of placental adhesion.

There is currently no clear recommendation for the treatment of high-grade placental abnormalities. In any case, early diagnosis and the creation of optimal (prepartum) conditions are crucial for clinical management [22,23].

## 11. Conclusions

The greatest risk factor for pathologic placentation is placenta previa following a previous cesarean section. The incidence of this condition is increasing due to rising rates of cesarean deliveries, resulting in higher maternal and neonatal morbidity and mortality. Prenatal diagnosis plays a crucial role in improving outcomes, and standardized ultrasound criteria should be employed for accurate prenatal diagnosis. Only with a correct tentative diagnosis can optimal management be achieved, including individualized planning, delivery at specialized centers, and care by interdisciplinary teams of experts.

Management of placenta accreta spectrum (PAS) requires a skilled surgeon, preferably trained in gynecologic oncology, and a multidisciplinary team. Rapid access to blood products and intensive care support is essential for successful care, particularly when a caesarean hysterectomy is indicated upon confirmation of PAS.

In cases where PAS is known preoperatively, uterotomy should be performed as far from the placental attachment site as possible. In severe cases, where detachment of the placenta may lead to uncontrolled bleeding, a caesarean hysterectomy en bloc (removal of uterus with placenta intact) is recommended.

Uterus-preserving approaches, such as leaving the placenta in situ for a wait-and-see approach or uteroplacental excision, should be considered only in carefully selected cases. In rare instances, a supracervical hysterectomy may be an alternative to complete caesarean hysterectomy, depending on individual circumstances. However, the decision for the most appropriate management strategy should be made based on the specific clinical scenario and with input from a specialized team of healthcare providers.

## Data Availability

The original contributions presented in the study are included in the article, further inquiries can be directed to the corresponding author.
